# Preprocessing NIR Spectra for Aquaphotomics

**DOI:** 10.3390/molecules27206795

**Published:** 2022-10-11

**Authors:** Jean-Michel Roger, Alexandre Mallet, Federico Marini

**Affiliations:** 1 ITAP, INRAE Montpellier Institut Agro, University Montpellier, 34196 Montpellier, France; 2 ChemHouse Research Group, 34196 Montpellier, France; 3 BioEnTech, 74 Av. Paul Sabatier, 11100 Narbonne, France; 4 Department of Chemistry, University of Rome “La Sapienza”, Piazzale Aldo Moro 5, 00185 Rome, Italy

**Keywords:** near infrared spectroscopy, preprocessing, aquaphotomics, chemometrics

## Abstract

Even though NIR spectroscopy is based on the Beer–Lambert law, which clearly relates the concentration of the absorbing elements with the absorbance, the measured spectra are subject to spurious signals, such as additive and multiplicative effects. The use of NIR spectra, therefore, requires a preprocessing step. This article reviews the main preprocessing methods in the light of aquaphotomics. Simple methods for visualizing the spectra are proposed in order to guide the user in the choice of the best preprocessing. The most common chemometrics preprocessing are presented and illustrated by three real datasets. Some preprocessing aims to produce a spectrum as close as possible to the absorbance that would have been measured under ideal conditions and is very useful for the establishment of an aquagram. Others, dedicated to the improvement of the resolution of the spectra, are very useful for the identification of the peaks. Finally, special attention is given to the problem of reducing multiplicative effects and to the potential pitfalls of some very popular methods in chemometrics. Alternatives proposed in recent papers are presented.

## 1. Introduction

Infrared spectroscopy is based on the phenomenon of absorption of photons by molecular bonds. The phenomenon of absorption obeys the Beer–Lambert law, which relates linearly the absorbance to the concentration (see Section 2). Each vibration of the molecular bonds, such as elongations or deformations, is responsible for a fundamental absorption [1]. These absorption bands are observed in the mid-infrared range (MIR, from 2.5 μm to 25 μm, corresponding to 4000–400 cm^−1^). However, near infrared spectroscopy (NIR, from 0.8 μm to 2.5 μm, i.e., 800 nm to 2500 nm, corresponding to 12,500–4000 cm^−1^), which contains the harmonics and combinations of the MIR bands, is by far the most used technique. One reason for this success is that the interaction between matter and NIR radiation is both weak and real [2]. Because it is weak, the photons can pass through a large amount of matter, typically several millimeters or even centimeters. Because it is effective, it results in an informative spectrum. However, the absorption peaks are weak and intricated. Moreover, measurements can be distorted by equipment drift, temperature/humidity changes, or sample presentation. The NIR spectra must, therefore, be processed by multivariate dedicated techniques, which constitute the toolbox of chemometrics. Among them, pretreatments are used to get closer to the Beer–Lambert theory and, thus, be able to apply linear calibration models.

Aquaphotomics is a new discipline [3,4], which appeared in the early 2000s. It studies water as a multiple-element medium, thus gaining the advantage of being described in a multi-dimensional space, such as that offered by NIR spectroscopy. NIR is typically used to estimate concentrations of chemical compounds, or to determine the membership of a sample in a group. In contrast to this use, aquaphotomics aims to use NIR spectroscopy to better understand the state of water and its interactions with other compounds and complex systems. For this purpose, the concept of aquaphotome is defined [3]. It is based on a set of absorbance values at different wavelengths, defining a water absorbance spectral pattern (WASP), usually presented by an aquagram [4]. The aquagram is a kind of radar graph representing the absorbance intensities at a set of wavelengths. As a consequence, aquaphotomics requires efficient tools to retrieve, on the one hand, the wavelength position of peaks and, on the other hand, relative absorbance intensities at several wavelengths. Preprocessing must, therefore, preserve the extraction of these two pieces of information, or even improve it.

This article proposes a critical review of the preprocessing methods classically used in chemometrics for NIR spectra, putting them in perspective of their use in the context of aquaphotomics studies.

## 2. Why to Preprocess NIR Spectra?

**Figure 1:**
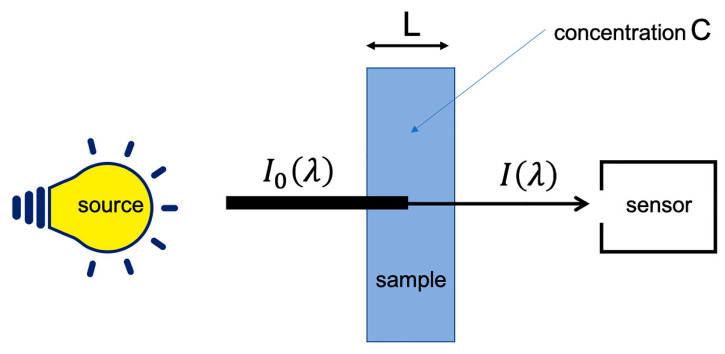
Ideal measurement of the absorption.

When electromagnetic radiation passes through a material medium, it undergoes several phenomena, including absorption. When the radiation is in the NIR range, this absorption is caused mainly by the bonds of nonsymmetrical molecules, including carbon, oxygen, hydrogen and nitrogen. This is why NIR spectroscopy is used in analytical chemistry of biological compounds. When a sample is measured under ideal conditions, i.e., in transmission, at low concentration of the analyte(s) of interest and without light scattering, Beer–Lambert law applies (see Figure 1 and Equation (1)).

(1) $$A_{0}\left(\lambda \right)=-log\left(\frac{I\left(\lambda \right)}{I_{0}\left(\lambda \right)}\right)=\varepsilon \left(\lambda \right)LC$$

where $A_{0}\left(\lambda \right)$ is the absorbance of the analyte at wavelength λ, $I\left(\lambda \right)$ and $I_{0}\left(\lambda \right)$ represent the intensity of the transmitted and incident light at the same wavelength, $\varepsilon \left(\lambda \right)$ is the molar absorptivity (molar extinction coefficient) of the chromophore, whose concentration is *C*, and *L* is the optical pathlength.

**Figure 2:**
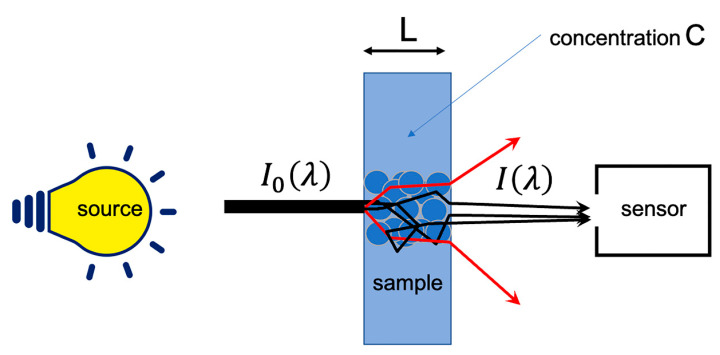
Real measurement of the absorption.

However, under current measurement conditions, a number of phenomena are added to the molecular absorption and Beer–Lambert’s law no longer applies. Thus, the interaction of radiation with particles and changes in optical index have the effect of modifying the path of photons. This results in a light scattering. This scattering has two consequences, illustrated by Figure 2. The first is a lengthening of the optical path, which introduces a multiplicative term. The second is a loss of photons, which will be falsely counted as an absorption and, thus, introduces an additive term. Some specific optical assemblies, using integrating spheres, allow getting rid of these phenomena. However, these systems require measurements in contact with the product (which may not always be possible), so that acquiring the signal simply in reflection mode is the option often preferred.

It is usually admitted that the multiplicative term does not depend on the wavelength and that the additive term does. This dependency can be modeled as a polynomial of λ of low degree (up to 2 or 3), and this additive term is, thus, called the baseline [5]. In turn, extra random and stochastic noise is added to the absorbance measurement, which finally results in Equation (2).

(2) $$A\left(\lambda \right)=\varepsilon \left(\lambda \right)kLC+A_{b}\left(\lambda \right)+A_{n}\left(\lambda \right)=kA_{0}\left(\lambda \right)+A_{b}\left(\lambda \right)+A_{n}\left(\lambda \right)$$

where k, $A_{b}\left(\lambda \right)$ and $A_{n}\left(\lambda \right)$ are a multiplicative factor, a baseline and a random noise, respectively.

When the measured spectra are used to estimate the concentration of a compound, by means of a calibration, or to explain variations related to this concentration, by means of an unsupervised analysis, the additive and multiplicative effects can be detrimental. Thus, in chemometrics, preprocessing aims to correct the experimentally measured absorbance, as expressed in Equation (2), so as to make the relation between the spectra and the concentration *C* more adherent to the postulated linear model [6,7]. Thus, preprocessing methods are dedicated to reduce or linearize the multiplicative and additive effects. In aquaphotomics, preprocessing aims to reveal a signal as close as possible to the real absorbance, as expressed in Equation (1). For this purpose, pretreatments that reveal hidden peaks and those that restore absorbance intensities are relevant.

## 3. Data

To illustrate the different preprocessing methods, three sets of data will be used along this paper:

- The first one contains 187 NIR spectra of virgin olive oils from Southern France measured in transmission [8]. The spectra were recorded at 612 wavelengths regularly spaced every 2 nm over the range 1000–2222 nm. The spectra were converted in absorbance.
- The second one contains 150 Vis-NIR spectra of grapes measured in transmission [9]. The spectra were recorded at 256 wavelengths regularly spaced every 3.30 nm over the range 303–1146 nm. No transformation was conducted on the spectra which are, thus, raw intensity spectra.
- The third one contains 126 NIR spectra of flour measured in reflectance mode [10]. The spectra were recorded at 209 wavelengths regularly spaced every 6.28 nm over the range 1118–2425 nm. A log transformation was conducted on the spectra which are, thus, expressed as pseudo absorbances (log(1/R)).

**Figure 3:**
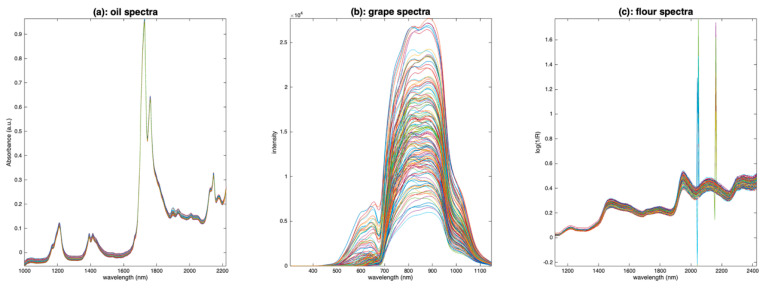
Spectra of olive oil (**a**), grapes (**b**) and flour (**c**).

The oil spectra (Figure 3a) are affected by a small additive effect. The grape spectra (Figure 3b) are affected by a huge multiplicative effect, due to the size of the measured berries. The flour spectra (Figure 3c) are affected by noise of two kinds: positive and negative spikes, between 2000 and 2200 nm, and uniform noise after 2200 nm. They also are affected by additive and/or multiplicative effects due to the scattering of the light into the flour.

## 4. Looking at the Data

It is important to look at the spectra before deciding which preprocessing to use. Several tools are available for this purpose.

### 4.1. Spectra Plot

**Figure 4:**
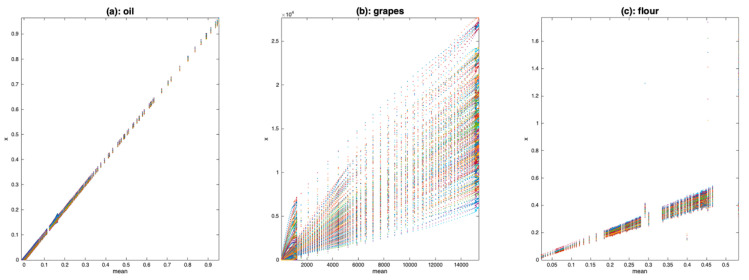
Scatter plot of the spectra as a function of the mean spectrum for (**a**): oil data, (**b**): grape data, (**c**): flour data.

One of the simplest visualization tools is to plot all the spectra, as a function of the average spectrum, using a scatter plot. Figure 4 shows the result of this visualization tool for the three datasets. Figure 4a clearly shows that the oil spectra are much more similar to one another than those of grapes (Figure 4b) or flour (Figure 4c). They differ by a small translation, corresponding to an additive effect not depending on the absorbance level. Figure 4b shows a very large variability between the spectra of grapes. All spectra are contained within a cone with the vertex at (0,0). This is characteristic of a pure multiplicative effect, due here to differences in the size of the measured berries. Figure 4c shows that the flour spectra are affected by isolated erroneous measurements, which dilate the vertical scale (spikes). Apart from these outliers, the flour spectra are organized in a cone but also appear to be affected by an additive effect. It is difficult to say whether these spectra contain a multiplicative effect or an additive effect increasing with wavelength.

### 4.2. PCA

**Figure 5:**
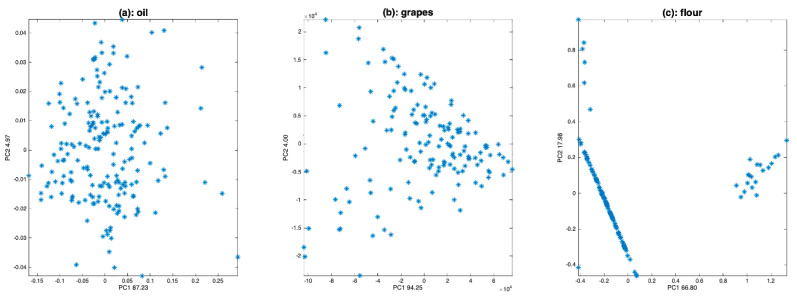
Scatter plot of the two first scores of a PCA calculated on (**a**): oil spectra, (**b**): grape spectra, (**c**): flour spectra.

**Figure 6:**
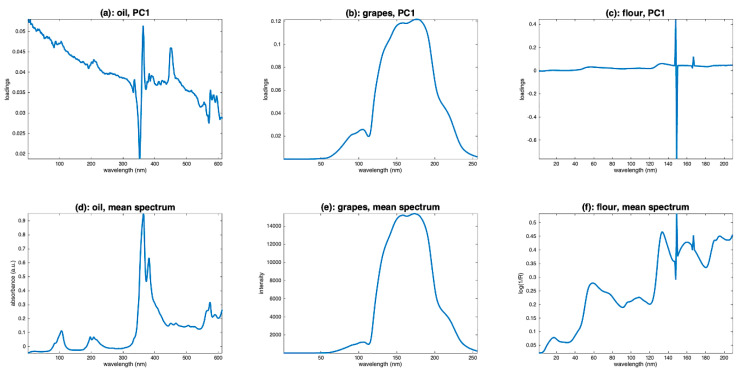
Loadings of a PCA calculated on (**a**): oil spectra, (**b**): grape spectra, (**c**): flour spectra, and mean spectra of (**d**): oil, (**e**): grape and (**f**): flour.

**Figure 7:**
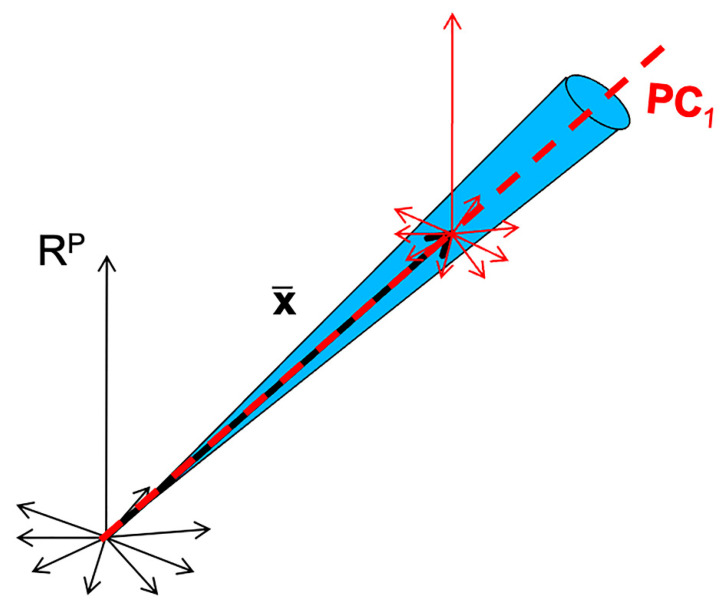
Illustration of the phenomenon of alignment between the mean spectrum and the first axis of the PCA in the case of the presence of multiplicative effects.

Another visualization tool is principal component analysis (PCA). It allows, by examining the scores, observation of the variability between the spectra. Examining the loadings also allows us to understand the source of the observed variability and to infer hypotheses about the presence of multiplicative or additive effects, and, thus, to guide the preprocessing step. Figure 5 shows the scores along the first two components of a PCA performed on the three sets of spectra. Figure 6 shows the corresponding loadings resulting from these PCA, together with the mean spectra of the three datasets. The scores of the grapes spectra (Figure 5b) show, from another point of view, the cone structure already observed in Figure 4b. In addition, the loadings on the PC1 axis (Figure 6b) are very similar to the mean spectrum (Figure 6e). Both observations are characteristic of a multiplicative effect. The reason for this is illustrated in Figure 7. It represents the spectra in the multi-dimensional wavelength space. When the spectra are impacted by a dominant multiplicative effect, they are organized in a very fine conical structure, passing through the origin of the space. When the data are centered, the canonical co-ordinate system is moved to the center of mass, at the end of the mean spectrum ($\bar{x}$). Then, the axis of greatest inertia (PC1) is calculated and, due to the shape of the spectra set, this axis is aligned with the mean spectrum. The score plot and the loadings of the flour spectra (Figure 6c,f) reveal that the presence of the spikes dominates. As a consequence, this dataset must be cleaned from these spikes before any other processing. The score plot and the loadings of the oil spectra (Figure 6a,d) do not reveal any particular structure.

## 5. Most Usual Preprocessing

### 5.1. Noise Removal

Noise corresponds to random variations in amplitude from one point to another in the spectrum. It is represented by the term $A_{n}\left(\lambda \right)$ of Equation (2). From a signal processing point of view, noise is considered as a high-frequency component. All NIR pre-processing dedicated to noise suppression is, therefore, based on low-pass filters, also called smoothing. In this section, the most commonly used smoothing methods are presented.

#### 5.1.1. Moving Averaging

A simple smoothing method is to replace the value of each point *i* in the spectrum by the average of the point values over a window of width *w*, centered on point *i*. The window is moved along the spectrum, hence the name moving average window or boxcar filter. This calculation cannot be applied to the first and last points of the spectrum. To overcome this problem, one can either extend the spectrum to the left and right before smoothing or keep the nonsmoothed points at both ends.

**Figure 8:**
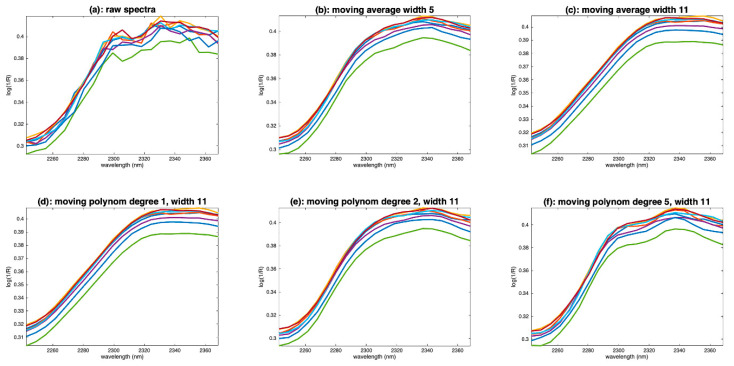
Examples of flour spectra in the wavelength range 2245–2368 nm. (**a**): Raw spectra, (**b**): spectra smoothed by a moving window average, width 5, (**c**): spectra smoothed by a moving window average, width 11, (**d**): spectra smoothed by a moving polynomial fitting, degree 1, width 11, (**d**): spectra smoothed by a moving polynomial fitting, degree 1, width 11, (**e**): spectra smoothed by a moving polynomial fitting, degree 2, width 11, (**f**): spectra smoothed by a moving polynomial fitting, degree 5, width 11.

Figure 8a shows the right-hand side of eight flour spectra for wavelengths above 2245 nm. We can notice the presence of noise, certainly due to the low sensitivity of the sensor often observed at the ends of the spectral range. Despite this noise, we can observe that the spectra show an increasing slope, from 2260 to 2320 nm, followed by a plateau until 2368 nm. This plateau is marked by a slight dip around 2315 nm and a decreasing slope from 2340 to 2368 nm. Figure 8b,c show the same spectra, once smoothed by a moving average of width 5 and 11, respectively. With a window of 5, the noise is not completely removed. The general shape of the spectra has been preserved, but the dip at 2315 nm has disappeared. The decreasing slope after 2340 nm has been preserved. With a window of 11, the noise is completely corrected, but the shape of the spectra has been dramatically altered. The increasing slope now ends at 2320 nm instead of 2300 nm; the dip at 2315 nm, as well as the decreasing slope after 2340 nm, have disappeared. We see on this example a limitation of the moving average smoothing method. When noise removal requires a window that is too wide, some low-frequency features may disappear, such as the dip at 2315 nm in our example. This limitation can be extremely problematic for aquaphotomics, which is based on the observation and measurement of such features.

#### 5.1.2. Moving Polynomial Fitting

To overcome this problem, it is possible to use a mobile polynomial smoothing. Mobile polynomial smoothing consists of identifying a polynomial of degree *d* in a window of width *w* centered on point *i*. The value of the point *i* is replaced by the value taken by the polynomial. The window is then moved by one point and the calculation is repeated. The same computational problem as for the moving average method arises at the ends of the spectra, and the same trick can be used to solve it. This method preserves the medium-frequency features modeled by the polynomial.

Figure 8d shows the result of smoothing using a window of width 11 and a polynomial of degree 1. We see the same problem as in Figure 8c. Figure 8e shows the result of smoothing with the same window width but using a polynomial of degree 2. We find a correct shape, with the main slope stopping at 2300 nm and the presence of the small decreasing slope after 2340 nm. On the other hand, the dip at 2315 nm is still absent. It is partially recovered by using a polynomial of degree 5, as shown in Figure 8f.

Moving window smoothing, irrespectively of whether it uses the average or the polynomial, is in fact a special case of the general filtering method of Savitsky and Golay, which will be detailed further.

#### 5.1.3. Frequency Filtering

By assimilating the wavelength scale to a time scale, another category of methods consists of decomposing the spectra on a frequency basis. These methods are based on transformations, such as Fourier or wavelet transforms. The signal is projected into a basis of signals of different frequencies. The higher-frequency components are eliminated, and the smoothed spectrum is obtained by the inverse transform. While the most common method to perform such a filtering is the Fourier transform, the wavelet transform is sometimes preferred for its ability to model complex shapes. These methods will not be further discussed here. The interested reader could find more details in [11].

#### 5.1.4. Median Filter

**Figure 9:**
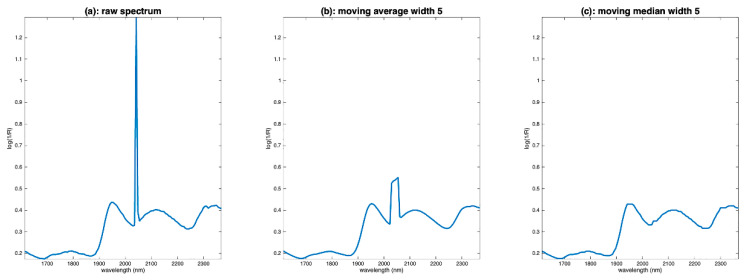
Illustration of the median filter on the first spectrum of the flour dataset. (**a**): Raw spectrum, (**b**): spectrum filtered by moving average, (**c**): spectrum filtered by moving median.

A special case of noise is in the form of peaks affecting a single wavelength, as in the example of flour spectra (Figure 3c). These peaks can originate from dead pixels in hyperspectral cameras. The elimination of such noise cannot be conducted using classical methods, such as moving averages. Instead, a median filter is used, which simply consists of replacing the value of the point i of the spectrum by the median of its neighbors. Figure 9a shows one spectrum of the flour database. We notice an intense spike around 2050 nm. Figure 9b shows the result of a moving average filtering on a window of five points. We can see that the filtering did not work because the noise is not zero mean. Figure 9c shows the result with a moving median filtering. The result is much better.

### 5.2. Baseline Removal

Baselines are low-frequency features added to the spectrum. They can be of different shapes. In NIR spectroscopy, baselines are due to light scattering, which causes a loss of photons arriving at the detector. They correspond to the term $A_{b}\left(\lambda \right)$ in Equation (2). They are almost always considered as polynomial functions of the wavelength. Thus, most of the preprocessing techniques dedicated to baseline removal use this model.

#### 5.2.1. Detrending

Since baselines are assumed to be low-order polynomial functions, a simple method of removing them is to fit the spectrum to a polynomial of a chosen degree and replace the spectrum with the residuals of the fit. This method, usually called “detrending”, is mathematically defined as follows [7]:

Let **v** be a column vector containing the wavelength values or even ${\left[1,\;2,\;\cdots ,p\right]}^{T}$, if the wavelengths are equally spaced. Let **V***_k_* be the (*p*, *k* + 1) matrix defined as follows:

$$V_{k}=\left[v^{0}v^{1}v^{2}\cdots v^{k}\right]=\left[\begin{array}{c} \begin{array}{ccc} 1 & \lambda_{1}^{\;} & \lambda_{1}^{2} \end{array}\\ \begin{array}{ccc} 1 & \lambda_{2}^{\;} & \lambda_{2}^{2} \end{array}\\ \begin{array}{ccc} \vdots & \vdots & \vdots \end{array}\\ \begin{array}{ccc} 1 & \lambda_{p}^{\;} & \lambda_{p}^{2} \end{array} \end{array}\;\begin{array}{c} \begin{array}{ccc} \ldots & & \lambda_{1}^{k} \end{array}\\ \begin{array}{ccc} \ldots & & \lambda_{2}^{k} \end{array}\\ \begin{array}{ccc} & & \vdots \end{array}\\ \begin{array}{ccc} \ldots & & \lambda_{p}^{k} \end{array} \end{array}\right]$$

If **X** contains the spectra to be detrended, the baselines contained in the rows of **X** are calculated by **V***_k_*:

$$L_{k}=X\;V_{k}{\left(V_{k}^{T}V_{k}^{\;}\right)}^{-1}V_{k}^{T}$$

and then removed from **X**:

(3) $$X_{det}=X-L_{k}=X-X\;V_{k}{\left(V_{k}^{T}V_{k}^{\;}\right)}^{-1}V_{k}^{T}$$

**Figure 10:**
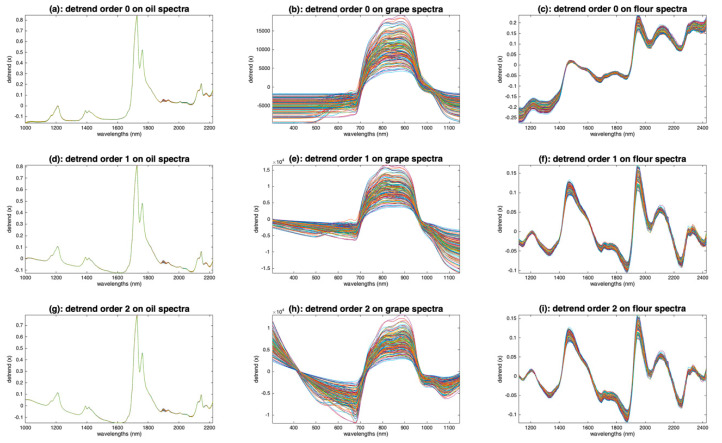
Illustration of the detrending preprocessing. (**a**,**d**,**g**): Oil spectra, (**b**,**e**,**h**): grape spectra, (**c**,**f**,**i**): flour spectra after median filtering (w = 3). (**a**–**c**): Order 0, (**d**–**f**): order 1 and (**g**–**i**): order 2.

Figure 10 shows the result of applying detrending with polynomial order 0, 1 and 2 on the oil, grapes and flour spectra. Compared with the raw oil spectra (Figure 3a), the detrended oil spectra have been cleaned from their inter-sample baseline variability. Already, by fitting a 0-order baseline (Figure 10a), we see that the spectra match perfectly, except after 1900 nm. This region, attributed to combinations of fundamental vibrations of C-H bonds, was identified by the authors of [7] as one of the most discriminating in the dataset. It can be seen that, with higher polynomial orders (Figure 10d,g), the parts without absorptions, before 1200 nm and between 1300 and 1400 nm, are no longer horizontal. In conclusion, for the oil spectra, a detrend of order 0 allows a very satisfactory shape to be obtained for the spectra. The application of detrending to the grape spectra (Figure 10b,e,h) does not give a good result. Indeed, the left and right parts of the spectra, which were naturally close to 0, are now completely shifted. In fact, regardless of the order of the polynomial used, detrending has added baselines to the spectra that originally contained none. This example illustrates how the application of preprocessing dedicated to additive effects is counterproductive when applied to spectra containing only multiplicative effects. Figure 10c,f,i show the application of detrending to the flour spectra. With a polynomial of degree 0 (Figure 10c), the baselines are corrected only in the center of the wavelength range. With a degree 1 (Figure 10f), the correction is better. The peaks at 1210 nm (fat), 1490 nm (starch), 1940 (water) and 2100 nm (proteins) are clearly exalted. It can be noticed that the use of a degree 2 (Figure 10i) does not bring anything better, which indicates that the baselines of the flour spectra are straight lines.

In chemometrics, detrending is often used because it removes sources of high variance, allowing the model to focus on useful information. In aquaphotomics, it allows the peaks to be highlighted, as can be seen on the example of the flour spectra, and it can also correct distortions that are detrimental to the construction of aquagrams.

#### 5.2.2. Derivatives

If the baselines are polynomials added to the spectra, a derivative of sufficient order will eliminate them. Thus, a baseline of degree 1 will be eliminated with a derivative of order 2. If $A\left(\lambda \right)=\varepsilon \left(\lambda \right)LC+A_{b}\left(\lambda \right)$ and if $A_{b}\left(\lambda \right)$ is a polynomial of degree *d*, then:

$$\frac{\partial^{d+1}\left(A\left(\lambda \right)\right)}{\partial \lambda^{d+1}}=\frac{\partial^{d+1}\left(\varepsilon \left(\lambda \right)\right)}{\partial \lambda^{d+1}}LC$$

**Figure 11:**
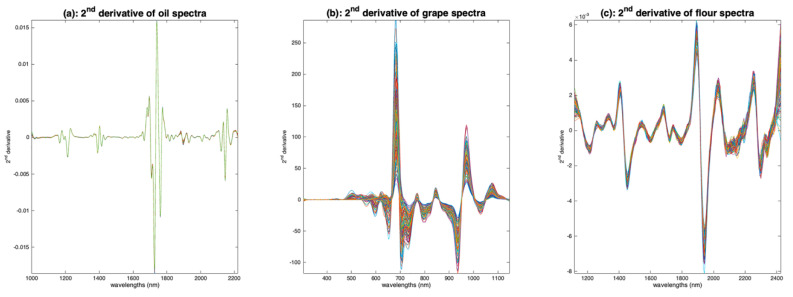
Illustration of the calculation of 2nd order derivatives by the Savitzky and Golay Algorithm. (**a**): Oil, (**b**): grapes, (**c**): flour. All the derivatives have been calculated using an 11 point window and a degree 2 polynomial.

To avoid increasing the noise of the spectra, the derivation operation must be performed with a particular algorithm. One of the most used algorithms in NIR spectroscopy is that of Savitzky and Golay [12]. To calculate the derivative at a point *i*, a polynomial of degree *d* is fitted to the points of a window centered on i and of width *w*; then, the polynomial is derived analytically and the value of the derivative at point *i* is adopted. Figure 11 shows the application of this algorithm to the three spectra sets, with a window of width 11, a polynomial of degree 3 and a derivative of order 2. One can see that all the base lines have disappeared and that a lot of peaks now appear.

The advantage of the derivatives is that the proportionality with the concentration is recovered. Moreover, this way of removing baselines acts locally, unlike detrending. Thus, if the baseline equation changes with wavelength, the derivation remains effective. The major disadvantage is that the spectrum shape is completely changed, because what appears in the derived spectrum is not the extinction spectrum $\varepsilon \left(\lambda \right)$ but its derivative.

However, this feature can be very advantageous for peak identification, and thus for aquaphotomics. For example, in [13], the second derivative of the water spectrum, calculated by the algorithm of Savitzky and Golay, allowed the identification of five species of water, differentiated by the H-bonding of their molecules. This method of pretreatment also allowed the authors to explore the behavior of these species when the temperature of the water changes. In [14], thanks to the peak deconvolution offered by second derivatives, the authors have put forward the existence of two peaks at 1412 nm and at 1462 nm corresponding to two OH-bond vibrational states. These spectacular results were obtained on very pure water. From our experience, second derivative can also reveal hidden peaks on more complex media where OH bond peaks are numerous and intricated, such as wet cellulosic products.

#### 5.2.3. Asymmetric Least Squares

Asymmetric least squares (ALS) allows complex baselines to be eliminated, while preserving the shape of the spectra [15]. It is a technique often used in Raman spectrometry to eliminate the fluorescence background. This method is actually a Whittaker filter, a standard signal processing tool. This method is based on the estimation of a spectrum **z**, approximating as well as possible the spectrum **x**, while having a smooth aspect. This is conducted by minimizing the expression:

$$\overset{\;}{\underset{i}{\sum }}\alpha_{i}{\left(x_{i}-z_{i}\right)}^{2}+\beta \overset{\;}{\underset{i}{\sum }}{\left(z_{i}-2z_{i}+z_{i+1}\right)}^{2}$$

where $\alpha_{i}$ is a weight assigned to each wavelength and β is a penalty term. Minimizing the first term of this sum tends to fit **z** to **x** and minimizing the second one tends to smooth **z**. In order to obtain positive smoothed spectra, the ALS idea is to calculate the weights as follows: $\alpha_{i}=q\;\mathrm{if}\;x_{i}>z_{i}$ and $\alpha_{i}=1-q$ otherwise. The two parameters β and q are user-defined and regulate the degree of smoothness of the estimated baseline and to what extent it is allowed to pass through the peaks.

**Figure 12:**
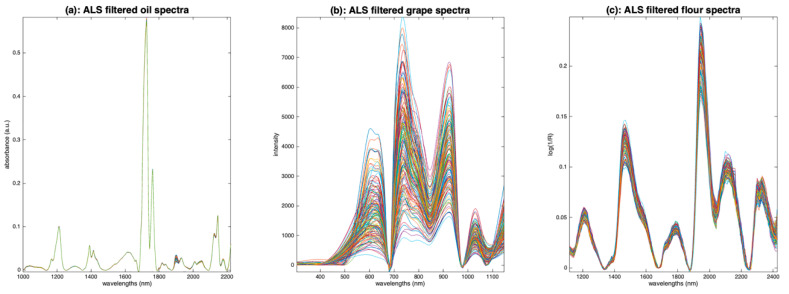
Illustration of asymmetric least squares filtering. (**a**): Oil, (**b**): grapes, (**c**): flour.

Figure 12 shows the results of applying ALS on the three sets of spectra. On the oil spectra (Figure 12a), the result is spectacular. The baselines have completely disappeared. All spectra are now based on a zero baseline and are positive, resembling pure absorbance spectra. The peaks appear clearly and so do the inter-sample variations. For the grape spectra (Figure 12b), the result is less convincing, certainly because this set of spectra is mainly affected by multiplicative effects. Nevertheless, the result is much better than the one obtained with detrending (Figure 10). The result on the flour spectra (Figure 12c) is very interesting. The baselines have disappeared, and the peaks and variations appear as clearly as with detrending (Figure 10). The advantage, compared to detrend, is that the spectra are positive.

The ALS pretreatment appears to be very relevant for aquaphotomics. Indeed, it produces positive spectra, with baselines at zero and clear peaks, which, in some cases, seem quite close to the ideal spectra.

### 5.3. Multiplicative Effect Removal

As expressed in Equation (2), a multiplicative effect is related to the factor k, which is assumed to multiply the measured values equally for all the wavelengths. This effect is very detrimental in chemometrics because it cannot be compensated by the linear models, contrarily to the additive effect. For this reason, preprocessing of multiplicative effects is often performed before calibration modeling of NIR spectra [16].

#### 5.3.1. Normalization

The basic idea of normalizing a spectrum, as explained in detail in [17], is to divide each of its variables by a quantity calculated from the spectrum and, if possible, affected only by the multiplicative effect. Formally, if $f\left(.\right)$ is this quantity, and using the expression in Equation (2) (omitting the additive terms), the normalization preprocessing can be written:

(4) $$A^{*}\left(\lambda \right)=\frac{kA_{0}\left(\lambda \right)}{f\left(kA_{0}\left(\lambda \right)\right)}=\frac{k\;A_{0}\left(\lambda \right)}{k\;f\left(A_{0}\left(\lambda \right)\right)}=\frac{\;1}{f\left(A_{0}\left(\lambda \right)\right)}A_{0}\left(\lambda \right)$$

We can notice in Equation (4) that the factor k has disappeared and that it has been replaced by a factor depending only on $A_{0}\left(\lambda \right)$. A side effect of this transformation can be to radically change the shape and physical meaning of the spectrum. Caution should, therefore, be paid when choosing the function f. A simple and intuitive choice for f consists of choosing a particular value of the spectrum, which is affected by k. Thus, the maximum of the spectrum is sometimes chosen. However, there are two problems with this choice: the first is that this point may be located on an absorption peak and, in this case, the normalization will remove some useful information. The second is that the wavelength of this point may change from one spectrum to another, which makes the normalization unstable. It is, therefore, preferable to choose, if it exists, a point in the spectrum with a large enough value but independent of the compound of interest. However, the value of a single point in the spectrum has some measurement noise. Dividing the spectrum by this value has the effect of increasing the overall noise of the spectrum. In order to avoid this phenomenon, it is preferable to use a function f calculated on the entire spectrum, such as the area, the mean, the sum, the norm or the standard deviation. When the spectrum contains only positive values, all these functions are almost equivalent. However, if the spectrum contains negative values, as, for example, if the spectrum has been differentiated before, the area, the sum or the mean can be close to zero and, thus, pose stability problems. For this reason, the most used function is the norm or the standard deviation.

**Figure 13:**
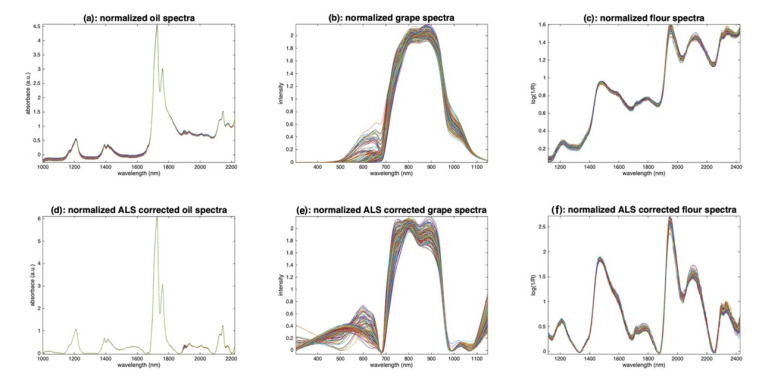
Illustration of normalization. (**a**,**d**): Oil, (**b**,**e**): grapes, (**c**,**f**): flour. (**a**–**c**): Raw spectra are divided by their norm. (**d**–**f**): Raw spectra are first corrected by ALS and then divided by their norm.

Figure 13a–c show the results of normalization by the norm for the three sets of spectra. The effect on the oil spectra (Figure 13a) is almost nonexistent, because these spectra contain no multiplicative effects. The application on the grape spectra is spectacular. The visible part of the spectrum, from 400 to 700 nm, shows several groups of different colors (with peaks at 550 nm or 600 nm), which corresponds well to the ground truth (i.e., different grape varieties). The VNIR part, from 700 to 1150 nm, is much less impacted by the multiplicative effect, although there is still some left. The peak at 960 nm, due to water, now appears more clearly. The flour spectra (Figure 13c) have also benefited from this normalization. The spectra are now much more clustered.

The explanations given, especially by Equation (4), assume that the spectra are free of additive effects. Figure 13d–f show the results of the normalization on the three sets of spectra after application of the ALS filter. It can be seen in Figure 13d that normalization did not alter the oil spectra, which were already near perfect after ALS filtering. On the other hand, the grape spectra, which were highly altered by ALS filtering (Figure 12b), do not give a good result after normalization. Finally, the application of normalization on the ALS-filtered flour spectra (Figure 13f) shows interesting results. The intensity variations that remained on the ALS-filtered spectra (Figure 12c) were significantly reduced. However, it can be seen that variations remain in areas where no chemical absorption takes place, such as between 1300 and 1400 nm. This can be explained by the fact that the spectrum norm, which is used in the normalization procedure, contains chemical information and is, therefore, not the best candidate for the function f. To avoid this problem, several methods have been proposed [17,18,19]. They all consist of estimating a function f related as much as possible to the multiplicative effect, and as little as possible to the chemistry of the sample.

#### 5.3.2. Probabilistic Quotient Normalization (PQN)

PQN proposes to estimate $f\left(x\right)$ on only a part of the wavelengths in order to avoid the problem mentioned above. It calculates the quotients of the values of all the variables of the spectrum x over those of the corresponding wavelengths in a reference signal $x_{\mathrm{ref}}$, thus producing as many divisors as wavelengths. Then, $f\left(x\right)$ is calculated as the most probable of these values, i.e., the mode of the divisor distribution. In practice, since it is often impossible to find the mode of a real distribution, the median is taken as an estimate of the most probable value. This method assumes that the variables affected only by the multiplicative effect are more numerous than those also affected by the compound of interest. This condition is rarely met in NIR spectroscopy applied to complex products but it could be met in some aquaphotomics applications.

**Figure 14:**
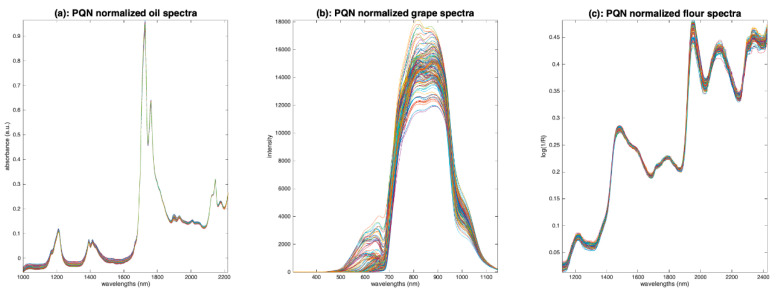
Illustration of probabilistic quotient normalization. (**a**): Oil, (**b**): grapes, (**c**): flour.

Figure 14a–c show the results of PQN for the three sets of spectra. Globally, as expected, the results are very similar to those of classical normalization (Figure 13).

### 5.4. Combined Methods

Methods have been developed to correct for additive and multiplicative effects simultaneously. This is the case of standard normal variate [20], which applies a detrend of order 0 and then a normalization by the standard deviation of the spectrum. This is a very popular method in the NIR community. SNV will be discussed in depth later, in Section 7.

Multiplicative scatter correction (MSC) [21] proposes explicitly correcting each spectrum x of a set of spectra X with respect to a reference spectrum $x_{ref}$. The idea is to remove from each spectrum the additive and multiplicative effects that distinguish it from $x_{ref}$ and, thus, to align all the spectra on a common global direction. In practice, as $x_{ref}$ is often unknown, it is usually replaced by the average of X. Concretely, using the point of view of Figure 4, the following model is written:

(5) $$x=ax_{ref}+b+r$$

Then, for each spectrum, a and b are derived by means of a linear regression, and x is corrected by:

(6) $$x_{msc}=\frac{x-b}{a}=x_{ref}+\frac{1}{a}r$$

In other words, each spectrum is modified so that it is as close as possible to the reference spectrum. Using the formalism of Equation (2), the idea is that all preprocessed spectra share the same k and $A_{b}\left(\lambda \right)\;\mathrm{as}\;\mathrm{the}\;\mathrm{reference}\;\mathrm{spectrum}$.

MSC is theoretically closely related to SNV [22]. It, therefore, suffers from the same problems as SNV (see Section 7). However, extended multiplicative scatter correction (EMSC) [5] differs from it by proposing more complex models. EMSC extends the MSC model by modeling the spectrum as a mixture of the reference spectrum and other spectral contributions, such as chemical interferents, temperature effects [23], optical scattering laws, etc. In this sense, EMSC is really a spectroscopic modeling method, as shown by the first sentence of [5]: “Knowledge-driven versus data driven modelling”. EMSC is, therefore, a tool of choice for aquaphotomics [24].

## 6. A Focus on Log Transform

While, in most cases, the additive and multiplicative effects are randomly distributed amongst the spectra, it was recently shown that these effects could be, in fact, directly related to the moisture content of the measured scattering media [25]. By using the EMSC framework to get rid of the additive effects, it could be shown that the path-length modifications (responsible of a multiplicative effect) can be directly related to moisture content by a simple power law. Coming back to Equation (2), this results in the following new equation:

(7) $$A\left(\lambda \right)=\varepsilon \left(\lambda \right)kLC+\varepsilon_{w}\left(\lambda \right)k_{w}L_{w}C_{w}^{p}+A_{b}\left(\lambda \right)+A_{n}\left(\lambda \right)$$

where all the variables subscripted by w relate to water, and *p* is the power coefficient.

In the specific case when moisture content is predicted, applying a log transformation of both the signal (after getting rid of the additive terms) and the predicted variable yields better models, as the log transform linearizes the relationship.

For a broader use, in aquaphotomics, when running multivariate curve resolution experiments, for example, the water effects on scattering could be included as a component, where a hard-modeling constraint could be applied on the shape of the component’s score/concentration (i.e., force the shape to be a power law type). Such information added as a constraint could help obtain a better resolution of the mixing problem.

## 7. A Focus on SNV

SNV is a very popular method in the NIR community; the paper describing this method has been cited more than 3400 times in about 30 years. The reason for such success is that the method is simple and effective in removing most of the variability due to scattering, which is unavoidable in case of backscatter measurements. It is widely used as a preprocessing for linear model calibrations, such as PLS regression. However, when the goal is to recover fundamental spectral components, such as pure spectra, as with $\varepsilon \left(\lambda \right)$ in Equation (1), or to recover an accurate level of absorbance, this preprocessing can be detrimental, as shown in the following example.

**Figure 15:**
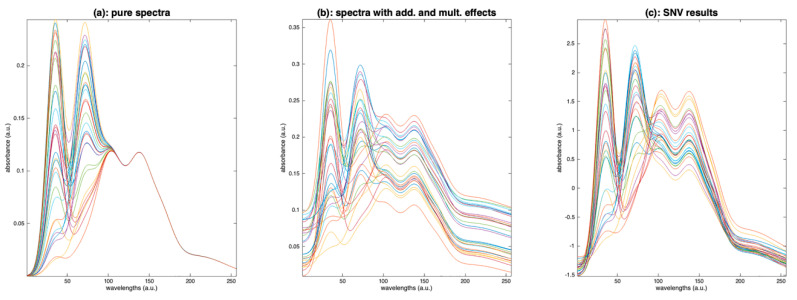
Illustration of the detrimental effect of SNV. (**a**) Simulated pure spectra, (**b**): simulated spectra after addition of baselines and multiplicative factors, (**c**): result of SNV performed on the spectra in (**b**).

Figure 15a represents a set of simulated spectra, where four peaks are present, with only the two left hand ones, related to the compounds of interest, varying. In Figure 15b, the same spectra have been assigned a baseline and a multiplicative effect, both independent of wavelength. Figure 15c shows the result of applying SNV on these spectra. The result is far from the pure spectra. For example, the variations at the two right peaks, which should be weak, are comparable to the ones of the peaks of interest. The problem is that SNV estimates the additive and multiplicative effects by the mean and standard deviation of the whole spectrum, respectively. However, these quantities, in our example (spectra in Figure 15b), are very largely influenced by the variations of the compound of interest. Thus, after applying the correction, the variations due to the compound of interest are distributed over the whole spectrum.

A solution consists of performing the estimation of the baseline and the multiplicative effect, preferentially using the wavelengths that are mainly related to the additive and multiplicative effects. This can be conducted using a diagonal matrix W of weights, between 0 and 1, which is then used in SNV and detrending:

- Compute the standard deviation of xW in place of x;
- Replace Equation (3) of detrending by: $x_{det}=x\left(\;I-W\;L\;{\left(L’\;W\;L\right)}^{-1}L’\;\right)$.

**Figure 16:**
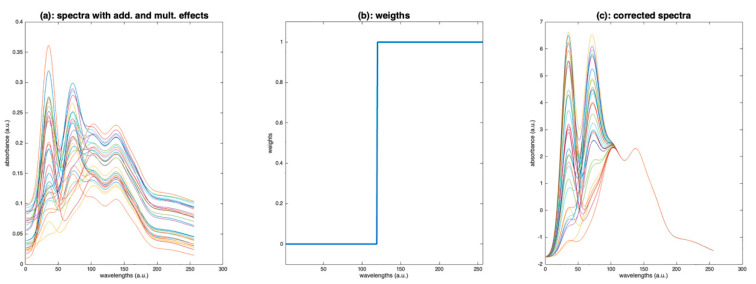
Illustration of the weighted SNV. (**a**): Spectra to be processed, (**b**): weights, (**c**): spectra after preprocessing by weighted SNV.

As an example, Figure 16c shows the result of a weighted SNV, using the weights plotted in Figure 16b. The result is perfect, because the additive and multiplicative effects have been estimated using the part of the spectra affected only by these effects.

The variable sorting for normalization (VSN) method [17] automatically calculates these weights **W** from the experimental data matrix **X**. It uses an RANSAC-type algorithm [26] to classify the points of each spectrum into two classes: the inliers are the wavelengths that all share the same additive and multiplicative effect pattern; the outliers are the wavelengths related to nonsystematic variations, thus related to the compounds of interest. The final weight is determined as the probability that each wavelength belongs to the inlier class. VSN also determines the best-suited degree of the polynomial for the baseline.

**Figure 17:**
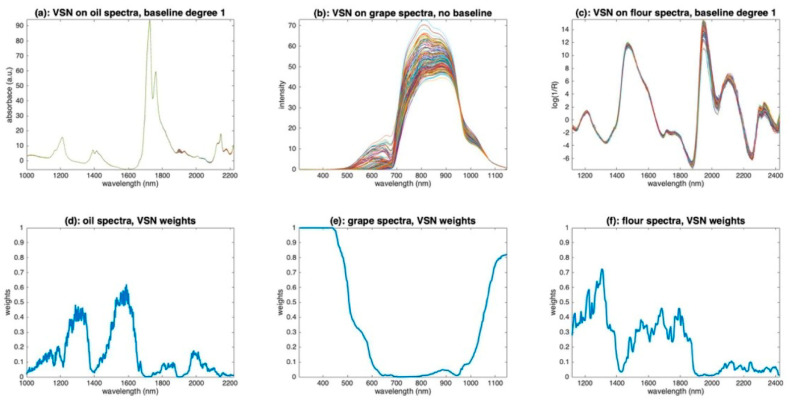
Illustration of the application of VSN. (**a**): VSN corrected oil spectra, (**b**): VSN corrected grape spectra, (**c**): VSN corrected flour spectra, (**d**): VSN weights for the oil spectra, (**e**): VSN weights for the grape spectra, (**f**): VSN weights for the flour spectra.

Figure 17 illustrates the application of VSN to the three sets of oil, grape and flour spectra. Figure 17a–c show the corrected spectra; Figure 17d–f show the weights found by the algorithm. For the oil spectra, VSN retained baselines of degree 1 and calculated the weights plotted in Figure 17d. These weights are overall low, indicating that all wavelengths are mostly related to chemistry. The two least informative areas, and, therefore, the most efficient for calculating additive and multiplicative effects, are the two dips around 1300 and 1500 nm. The resulting spectra reported in Figure 17a are very similar to those yielded by a detrending of order 1 (Figure 10d). For the grape spectra, VSN has identified that only multiplicative effects exist. The weights (Figure 17e) are very contrasting. They are very close to 1 at both ends of the spectral range, i.e., before 500 nm and after 1100 nm, and close to 0 in the 550–1000 nm region, which corresponds well to the region of pigments and metabolic compounds. The resulting spectra (Figure 17b) are quite similar to those obtained with PQN (Figure 14b). This illustrates that, when the spectra are only impacted by multiplicative effects, VSN and PQN are, in fact, two very close solutions to achieve a robust normalization. For the flour spectra, VSN found a model with baselines of degree 1, which corresponds to the result found after applying detrending (Figure 10). The weights (Figure 17f) show that the areas around 1400 nm and above 2000 nm should not be used for the calculation of multiplicative and additive effects. This result is consistent with the absorption areas of O-H bonds (water, cellulose) and proteins. The preprocessed spectra (Figure 17c) globally resemble those obtained with detrending (Figure 10). However, in detail, it can be seen that the area from 1900 nm to 2500 nm was less shrunk by the preprocessing than the rest of the spectrum, suggesting that variations due to chemistry are visible in this area.

It is clear that weighted versions of EMSC or SNV (thanks to PQN or VSN) appear most suitable for aquaphotomics studies. Indeed, while aquaphotomics focuses on the first O-H overtone region (1300–1600 nm), the preprocessing of physical effects should be conducted based on regions where absorbance bonds related to chemical phenomena are absent (in water spectra, this usually corresponds to the following spectral ranges: 1000–1300 nm, 1600–1800 nm, and 2000–2400 nm).

## 8. Conclusions

This paper has reviewed the main preprocessing of NIR spectra, with a view to their use in aquaphotomics. Simple visualization methods have been proposed in order to identify the nature of undesirable effects, even before the application of a preprocessing. The most commonly used preprocessing in chemometrics has been presented and illustrated by three sets of spectra, each showing the advantages and disadvantages of each method. Some preprocessing aims to produce a spectrum as close as possible to the absorbance that would have been measured under ideal conditions and can, therefore, be very useful for the establishment of an aquagram. Others, dedicated to the improvement of the resolution of the peaks, prove to be very useful for the identification of hidden peaks. A special focus on the correction of multiplicative effects has shown the interest of logarithmic transformation and weighted normalization methods. This highlights the fact that much preprocessing that has been designed in chemometrics to improve the performance of calibration models should be used with care in aquaphotomics.
